# High Resolution Mapping of QTLs for Heat Tolerance in Rice Using a 5K SNP Array

**DOI:** 10.1186/s12284-017-0167-0

**Published:** 2017-06-05

**Authors:** Shanmugavadivel PS, Amitha Mithra SV, Chandra Prakash, Ramkumar MK, Ratan Tiwari, Trilochan Mohapatra, Nagendra Kumar Singh

**Affiliations:** 1 ICAR-National Research Centre on Plant Biotechnology, New Delhi, India; 2 Present address, Division of Plant Biotechnology, ICAR-Indian Institute of Pulses Research, Kanpur, 208 024 India; 3 ICAR-Indian Institute of Wheat and Barley Research, Karnal, 132 001 India; 4 Indian Council of Agricultural Research, Krishi Bhavan, New Delhi, 110 001 India

**Keywords:** Rice, Heat tolerance, Nagina22, QTL mapping, SNP

## Abstract

**Background:**

Heat stress is one of the major abiotic threats to rice production, next to drought and salinity stress. Incidence of heat stress at reproductive phase of the crop results in abnormal pollination leading to floret sterility, low seed set and poor grain quality. Identification of QTLs and causal genes for heat stress tolerance at flowering will facilitate breeding for improved heat tolerance in rice. In the present study, we used 272 F_8_ recombinant inbred lines derived from a cross between Nagina22, a well-known heat tolerant *Aus* cultivar and IR64, a heat sensitive popular *Indica* rice variety to map the QTLs for heat tolerance.

**Results:**

To enable precise phenotyping for heat stress tolerance, we used a controlled phenotyping facility available at ICAR-Indian Institute of Wheat and Barley Research, Karnal, India. Based on ‘days to 50% flowering’ data of the RILs, we followed staggered sowing to synchronize flowering to impose heat stress at uniform stage. Using the Illumina infinium 5K SNP array for genotyping the parents and the RILs, and stress susceptibility and stress tolerance indices (SSI and STI) of percent spikelet sterility and yield per plant (g), we identified five QTLs on chromosomes 3, 5, 9 and 12. The identified QTLs explained phenotypic variation in the range of 6.27 to 21. 29%. Of these five QTLs, two high effect QTLs, one novel (*qSTIPSS9*.1) and one known (*qSTIY5.1/qSSIY5*.2), were mapped in less than 400 Kbp genomic regions, comprising of 65 and 54 genes, respectively.

**Conclusions:**

The present study identified two major QTLs for heat tolerance in rice in narrow physical intervals, which can be employed for crop improvement by marker assisted selection (MAS) after development of suitable scorable markers for breeding of high yielding heat tolerant rice varieties. This is the first report of a major QTL for heat tolerance on chromosome 9 of rice. Further, a known QTL for heat tolerance on chromosome 5 was narrowed down from 23 Mb to 331 Kbp in this study.

**Electronic supplementary material:**

The online version of this article (doi:10.1186/s12284-017-0167-0) contains supplementary material, which is available to authorized users.

## Background

Rice is a major staple food crop for nearly half of the world population. The global population is projected to grow from seven to nine billion by 2050 and to reach ten billion before 2100 (United Nations 2011). To ensure food security to the added population, rice production has to increase by 0.6 to 0.9% annually until 2050 (Carriger and Vallee 2007). However, rise in global average temperature to the tune of 0.5 °C in the twentieth century and future projections in the range of 1.4–5.8 °C by the end of this century (IPCC 2007), will be detrimental to crop yield (Lobell et al. 2011). Declining farmland resources coupled with global warming have forced rice cultivation to marginal environments and beyond the normal rice season. This in turn has exposed the rice crop to higher day temperature (>33 °C) adversely impacting seed set (Nakagawa et al. 2002; Prasad et al. 2006; Jagadish et al. 2010a).

Heat stress alters the initiation and duration of developmental phases, especially the duration from floral/panicle initiation to anthesis/panicle exertion in plants (Sato et al. 1973). Heat stress during flowering and anthesis can lead to failure in fertilization due to pollen or ovule sterility (Matsui et al. 1997). Early reproductive processes viz., micro- and megasporogenesis, pollen and stigma viability, anthesis, pollination, pollen tube growth, fertilization, and early embryo development are all highly susceptible to heat stress (Giorno et al. 2013). Flowering stage is crucial for crop productivity as heat stress during this phase causes reduced pollen fertility and low seed set in rice (Jagadish et al. 2010a). Anthesis processes, including anther dehiscence, pollination, and pollen germination are most sensitive to high temperature stress in rice. The main cause of spikelet sterility induced by high temperature is anther indehiscence (Matsui et al. 1999). High temperature inhibits swelling of pollen grains, which is a driving force for anther dehiscence in rice. Successful anther dehiscence depends on several parameters, including rupturing of septa, expansion of locule walls, pollen swelling, and rupturing of stomium (Liu et al. 2006).

Enhanced heat tolerance in rice is required at flowering stage to avoid spikelet sterility. Since 1985, germplasm screening for high temperature stress tolerance has been carried out by different research groups worldwide (Sarwar and Avesi 1985; Matsui and Omasa 2002; Jagadish et al. 2007, 2008). Heat tolerance at flowering stage in rice is attributed to multiple genes with cumulative effects on trait expression, otherwise called as quantitative trait loci (QTL; Cao et al. 2003; Xiao et al. 2011a; Ye et al. 2012). The discovery of genes/QTLs for enhanced tolerance to high temperature stress has practical implications in agriculture. Mapping of QTLs for heat tolerance in rice was first reported by Cao et al. (2003) based on percent spikelet fertility using doubled haploid population derived from IR64/Azucena cross. Thereafter many research groups have mapped QTLs for heat stress tolerance using F_2_, back cross inbred lines (BIL) and recombinant inbred lines (RIL) populations, evaluated at the time of heading in controlled environment conditions (Chang-Lan et al. 2005; Chen et al. 2008; Zhang et al. 2008, 2009; Jagadish et al. 2010b; Xiao et al. 2011a; Ye et al. 2012, 2015; Cheng et al. 2012; and Poli et al. 2013). Some of these studies created high temperature condition for phenotyping by late planting in open field (Xiao et al. 2011b; Tazib et al. 2015; and Zhao et al. 2016). Almost all of these studies employed RFLP or SSR markers, except Ye et al. (2012, 2015), who used 300 SNP markers for the QTL mapping. IR64 has been used as one of the parental lines in generating mapping populations for mapping heat stress tolerance QTLs in some studies (Cao et al. 2003; Ye et al. 2012, 2015), while Nagina22 and its derived mutant lines have been used as parents in generating mapping population by other researchers (Buu et al. 2014; Poli et al. 2013). There is a report on using IR64/Nagina22 derived F_2_ population for mapping heat tolerance QTLs (Ye et al. 2012).

Mapping of QTLs for heat stress tolerance using stress tolerance indices, which compare the performance of genotypes under control and stress condition, have not been reported earlier, but it has been utilized for mapping salt stress tolerance (Fernandez 1992; Pandit et al. 2010; Tiwari et al. 2016). The relative performance of genotypes under stress and control conditions can be used as an indicator to identify and map QTLs, which can be further used in breeding crop varieties for stress tolerance, rather than mapping QTLs based on phenotypic performance in stress environment alone (Raman et al. 2012). This has practical relevance since genotypes with low yield potential under control condition quite often show higher tolerance to stress than high yielding genotypes. Genomic regions governing salinity stress tolerance was successfully mapped in rice using stress indices (Pandit et al. 2010, Kumar et al. 2015; Tiwari et al. 2016). The present study focused on identification of QTLs for heat stress tolerance at flowering stage in a RIL mapping population derived from Nagina22/IR64 cross using controlled phenotyping facility for imposing heat stress, using stress tolerance indices for normalization of intrinsic differences in yield potential and high density SNP mapping. High density linkage map is expected to result in finding QTLs flanked by closely linked markers that can be readily used in breeding programmes for marker assisted selection.

## Methods

### Plant Materials

We used 272 F_7:8_ RILs developed through single seed descent method from a cross between Nagina22 (N22), a heat tolerant cultivar (Mohapatra et al. 2014; Prakash et al. 2016) and IR64, a heat susceptible cultivar (Jagadish et al. 2010). To achieve synchronized flowering, the RILs were first phenotyped for days to 50% flowering and grouped into three categories as early, medium and late flowering types and then were sown in a staggered manner for synchronization of their flowering time. This exercise enabled us to impose heat treatment at a uniform stage in the population that in turn minimized the interference of phenological differences in analysis.

### Heat Stress Treatment

**Fig. 1:**
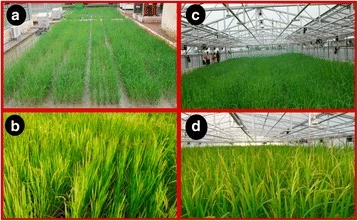
Phenotyping of F_8_ RIL mapping population under non-stress and heat stress conditions at ICAR-IIWBR. (**a**) At vegetative stage under non-stress condition (**b**). At reproductive stage under non-stress (**c**). At vegetative stage under non-stress condition inside controlled environment structure with roof top open (**d**). With reproductive stage heat stress for 10 days in controlled environment structure with roof top covered

The RILs along with the two parental lines were direct sown in the controlled temperature phenotyping facility at ICAR-Indian Institute of Wheat and Barley Research, Karnal, India in an augmented design for exposing them to high temperature at flowering stage. A plant-to-plant distance of 15 cm and row-to-row distance of 20 cm was maintained. The structure was kept open from sowing to till the flowering stage, where the experimental RILs were grown in a condition similar to that prevailing outside the green house (Fig. 1). Heat stress was imposed on plants during flowering time by closing the shutter. The temperature inside the structure was programmed to be 5 °C higher than the air temperature outside the structure (Additional file 1: Figure. S1). Relative humidity of 70% was maintained inside the facility. Heat stress was imposed continuously for 10 days including night time. After the treatment, the structure was kept open until harvest. The same RIL population was also raised outside the green house to phenotype their performance under control or ambient conditions.

### Phenotyping of the F_8_ RILs for Heat Stress Tolerance

Five individual plants from each RIL were sampled and harvested separately. Main panicle from each plant was used for analysing spikelet fertility by counting the number of filled and empty spikelets. The remaining panicles were collected separately from each plant and utilised for calculating yield potential of RILs under heat stress. In the same way, five plants from each RIL were harvested from the control experiment for evaluating the performance of genotypes under ambient conditions, used for computing their stress response index. The response of genotypes to heat stress was expressed as stress susceptibility index (SSI) given by Fisher and Mauer (1978) and stress tolerant index (STI) given by Fernandez (1992). SSI assesses the reduction in yield caused by stress as compared to favourable environment. The heat tolerant genotypes would have lower SSI value, which indicates lower difference in their yield across control and stress while it would be vice-versa for the susceptible genotypes. Thus, SSI helps to identify more tolerant lines. STI, on the other hand, helps to identify genotypes that produce higher yield in control as well as stress conditions, which is more desirable for practical reasons. The tolerant lines have higher STI value.

### Genotyping of RILs

Genomic DNA of all RILs, and the two parents was isolated from pooled young green leaves from plants grown in a row in the field by CTAB method (Doyle and Doyle 1990). DNA was quantified using NanoDrop 8000 spectrophotometer (Thermo Scientific, USA) and concentration of DNA was adjusted to a minimum of 50 ng/μl and approximately 200 ng of DNA from each genotypes were used for hybridization in Illumina Infinium® II genotyping assay. A customized array with 5246 SNPs in abiotic stress responsive genes of rice employing Illumina Infinium® II design probes and dual color channel assays (Infinium HD Assay Ultra, Illumina), was used for genotyping, following the manufacturer’s protocol (Kumar et al. 2015).

### SNP Genotype Calling

SNP genotyping data obtained from the array were analysed using Genome Studio V2010.1 (Illumina Inc.). SNPs were called using genotyping module integrated in the software where individual SNP is viewed as GenoPlots. Data quality was confirmed with internal controls and QC functions such as GenTrain and GenCall scores. After calling the data automatically, the SNPs were re-scored and checked for their presence in a canonical cluster to get a GenTrain score > 0.7. The samples with call rates of <0.89 and the SNPs with norm *R* values <0.2 were removed from further analysis.The genotype calls from parental lines Nagina22 and IR64 were converted into AA and BB, respectively and similarly SNP calls of segregating genotypes were transformed in concordance with either parental type and used for the construction of framework linkage map.

### QTL Mapping and Epistatic Interaction Network

QTL mapping was carried out using QTL IciMapping software v4.0 (Meng et al. 2015). Segregation pattern of each SNP in the RIL population was analysed using chi-square test with statistical significance at *P* value of 0.01. The redundant markers with identical scores were removed since they cannot provide any additional information. Markers with correlation coefficient of 1 were deleted by choosing missing proportion option. The genetic distance (cM) between SNP markers was converted to physical distance (kb) with 1 cM equal to 260 kbp (Chen et al. 2002; Tiwari et al. 2016). QTLs for heat stress tolerance were mapped using BIP functionality available in the QTL IciMapping software. Inclusive composite interval mapping of additive and dominant QTL (ICIM-ADD) mapping method was chosen along with the following parameters viz.*,* window size of 1 cM, 500 permutations and type I error at 0.05 to call for QTL. The LOD threshold was set at 3.5 to accept the call as a significant QTL. The epistatic interaction network was determined for all the four traits studied using MQM algorithm in R/QTL (Browman et al. 2003).

### *In-silico* Identification of Non-synonymous SNPs in Genes Present in the Mapped QTL Intervals

Non-synonymous SNPs between N22 and IR64 were identified using Rice SNP-Seek Database (Mansueto et al. 2017) with Nipponbare as reference genome and N22 and IR64 as query genomes. The gene locus id was given as input to retrieve the non-synonymous SNPs between N22 and IR64 for further analysis.

## Results

### Phenotypic Variation and Correlation Among Traits in the RIL Population

**Fig. 2:**
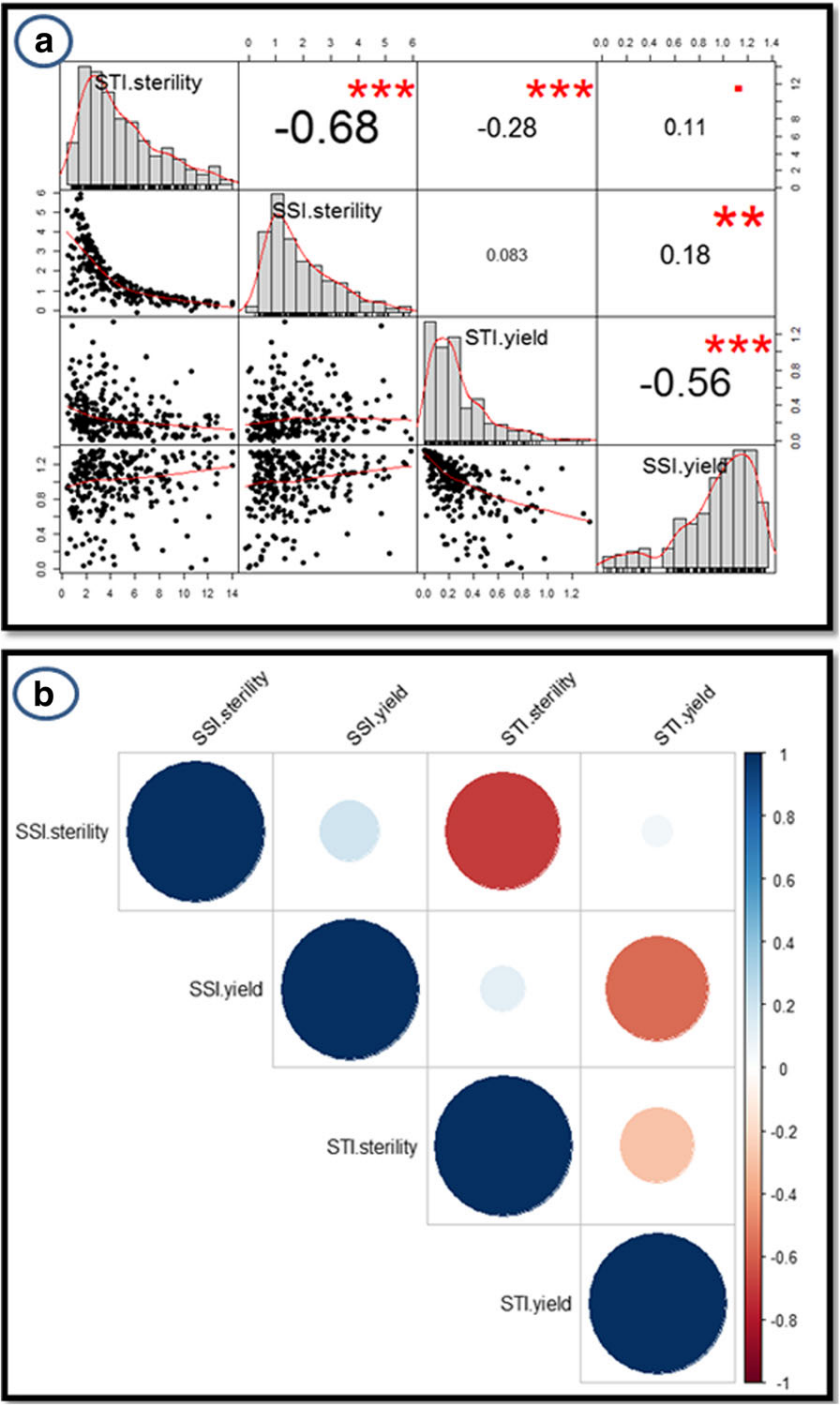
Phenotype distributions and Correlation of stress indices. (**a**) Trait distribution and linear correlation values (**b**). Correlogram of stress indices

The parents and RILs were analysed for their phenotypic performance under ambient and heat stress environments. Heat sensitive parent IR64 showed very high spikelet sterility (95.94%) under heat stress as compared to the tolerant parent N22 (67.45%; Table 1). The yield reduction under heat stress was to the tune of 66% in N22 while it was 86% in IR64. Though the RIL population mean for percent spikelet sterility and yield was skewed towards IR64, it showed transgressive segregation and had high coefficient of variation (CV), more than 20% under both control and stress conditions (Table 1). Since SSI and STI are better indicators of plant performance under stress, we used these indices for mapping QTLs for heat tolerance. The RIL population exhibited transgressive segregation for all the four parameters analysed, namely SSI and STI of both percent spikelet sterility and yield per plant. The STI for percent spikelet sterility ranged from 0.38 to 14 while SSI for percent spikelet sterility ranged from −0.11 to 5.92. Similarly, the STI for yield ranged from 0.005 to 1.34 while SSI for yield ranged from 0.0078 to 1.37 (Table 2). High CV was observed for all the traits in a range of 0.32 (SSI for yield per plant) to 0.85 (STI of yield per plant), suggesting that all the four traits were suitable for QTL mapping. As expected, significant negative correlation was observed between STI and SSI for percent spikelet sterility and, STI for percent spikelet sterility and STI for yield (Fig. 2). The only positive correlation was between SSI for percent spikelet sterility and SSI for yield per plant, which is expected.

**Table 1:** Performance of the parents and their recombinant inbred lines under control and heat stress

	Percent spikelet sterility		Yield per plant (g)	
	Control	Heat stress	Control	Heat stress
Nagina22	6.38	67.45	6.41	2.18
IR64	14.53	95.94	9.48	1.33
RILs	2.17–78.01	15.40–100.00	1.89–24.12	0.064–12.65
Mean	16.56	81.8	9.47	2.65
Range	75.84	84.60	22.23	12.59
SD^a^	12.85	16.4	3.19	2
CV^b^	77.60	20.05	33.7	75.5

^a^Standard Deviation

^b^Coefficient of variation

**Table 2:** Phenotypic variation for heat stress tolerance indices in Nagina22/IR 64 RIL mapping population

S. No.	Traits	RILs						N22	IR64
		Minimum	Maximum	Range	Mean	SD^a^	CV^b^		
1	SSI for % spikelet sterility	−0.10919	5.9246	6.03379	1.6576	1.2916	0.77919	2.422	1.418
2	STI for % spikeletsterility	0.38193	13.9979	13.6159	4.5501	3.1156	0.68473	1.576	5.106
3	STI for yield per plant	0.00469	1.33855	1.33386	0.2805	0.2395	0.85383	0.3314	0.066
4	SSI for yield per plant	0.00782	1.37249	1.36467	0.9764	0.3084	0.31585	0.9142	1.1918

^a^Standard Deviation

^b^Coefficient of variation

### SNP Marker Segregation and Framework Linkage Map

**Fig. 3:**
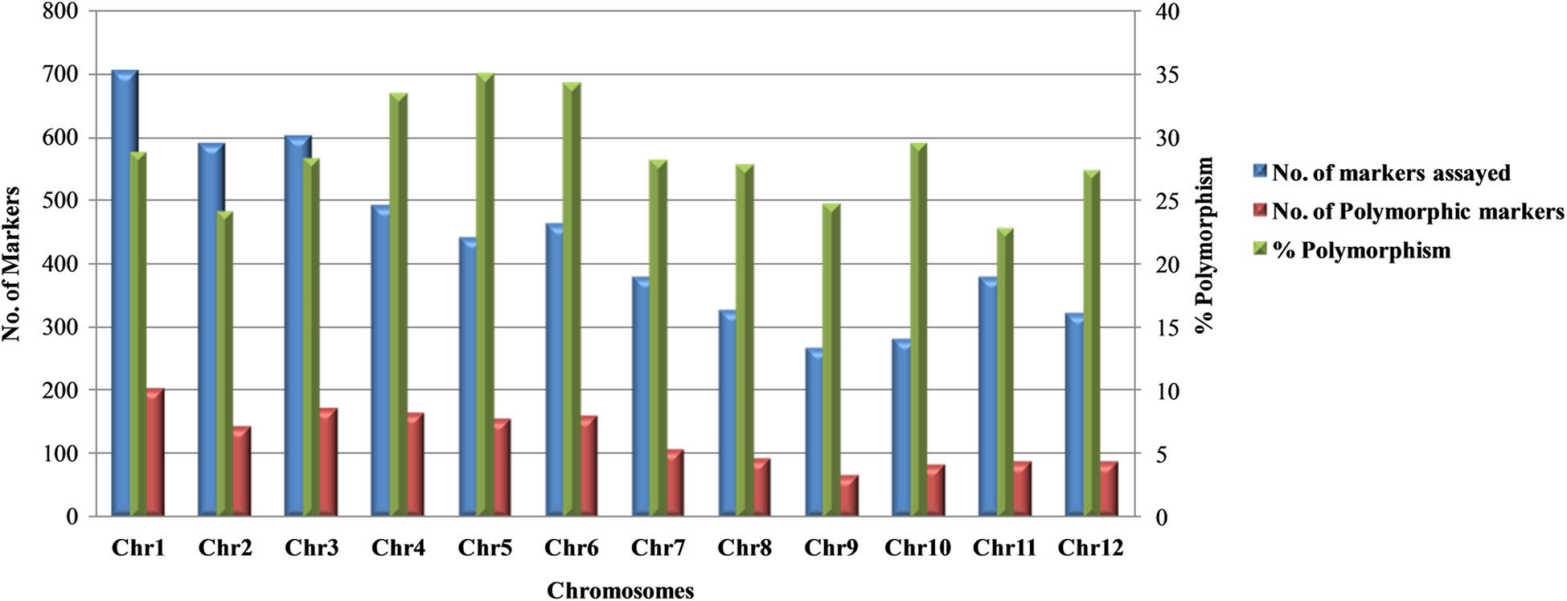
Chromosome-wise polymorphism survey using 5K SNP array and polymorphic SNPs in Nagina22 x IR64 mapping population

Out of the 5246 SNPs in the stress responsive genes genotyped using Illumina Infinium chip, 1512 were polymorphic between N22 and IR64 and segregated in the RIL population (28.82% polymorphism; Fig. 3). The highest number of polymorphic markers was present on chromosome 1 (203 SNPs) while the lowest was on chromosome 9 (66). In terms of proportion of polymorphic markers, chromosome 5 had the highest (0.35) proportion while chromosome 11 had the least (0.23). Thirty eight percent of polymorphic markers did not segregate as per the expected Mendelian segregation of 1:1 at cut-off probability of 0.01 and hence they were removed from further analysis. Another 117 markers that were redundant and played no role in improving the resolution of genetic map were also removed from further analysis. The highest number of redundant markers was found on chromosome 5 (24.76%) while the lowest number was on chromosome 8 (3.92%; Table 3). After removing the redundant markers, Chromosome 1 had the maximum number of markers (127) while chromosomes 4 and 7 had the lowest number of 31 markers. Finally, 824 markers were included in the framework linkage map used for QTL mapping.

**Table 3:** Selection of polymorphic markers for QTL mapping

	Chr1	Chr2	Chr3	Chr4	Chr5	Chr6	Chr7	Chr8	Chr9	Chr10	Chr11	Chr12	Total
No. of Markers after Chi square test at 0.01 significant level	152	120	94	33	105	108	34	51	51	63	62	68	941
% Segregation distortion	25.12	15.49	44.7	79.87	31.81	31.64	68.22	43.95	22.72	24.09	27.9	22.72	37.76
No. of markers per chromosome after removing redundant markers	127	107	83	31	79	98	31	49	43	58	58	60	824
% Redundant markers	16.44	10.83	11.7	6.06	24.76	9.26	8.82	3.92	15.68	7.93	6.45	11.76	12.43
No. of markers used for QTL analysis	127	107	83	31	79	98	31	49	43	58	58	60	824

**Fig.4:**
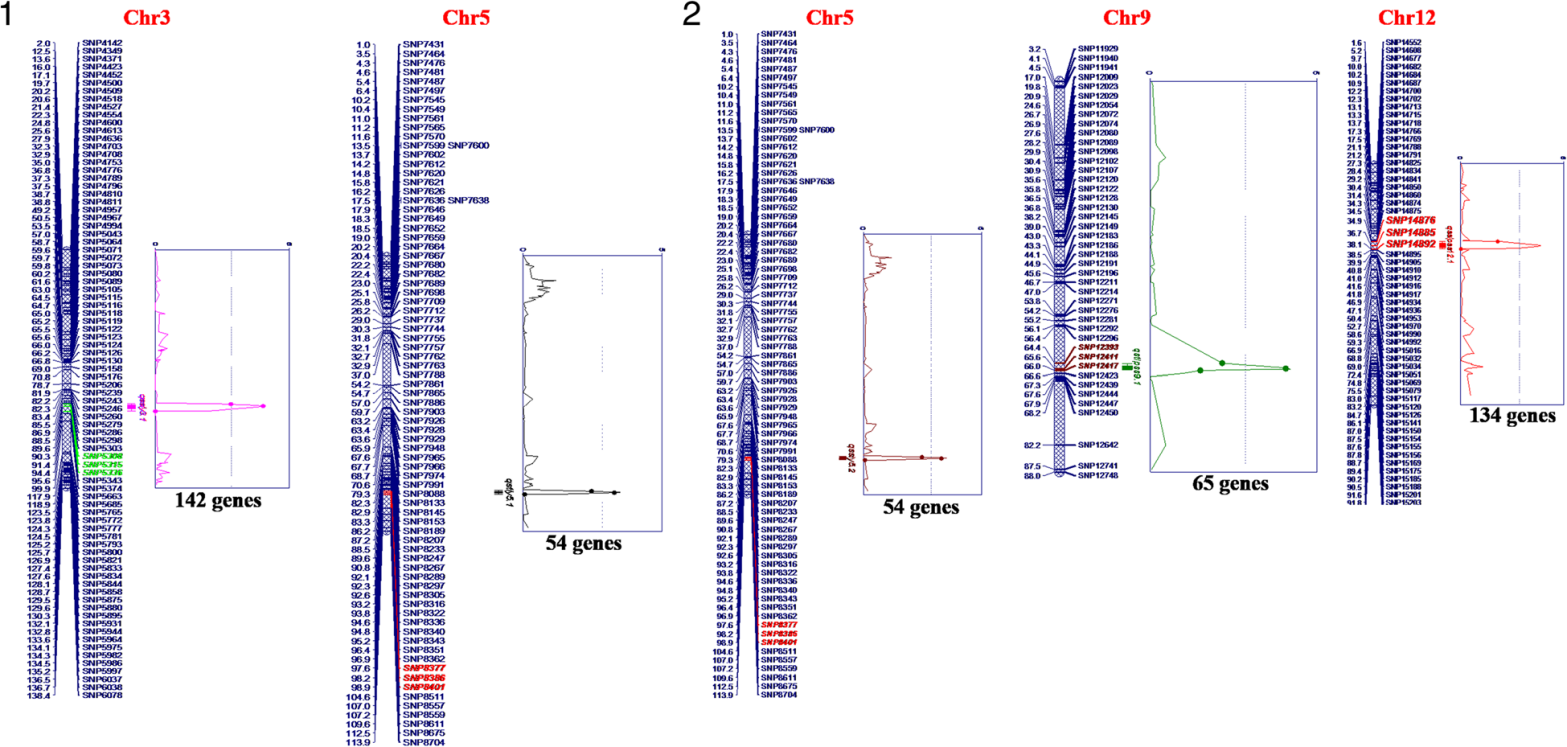
QTLs identified for heat tolerance in rice in the mapping population derived from Nagina22 and IR64

**Fig. 5:**
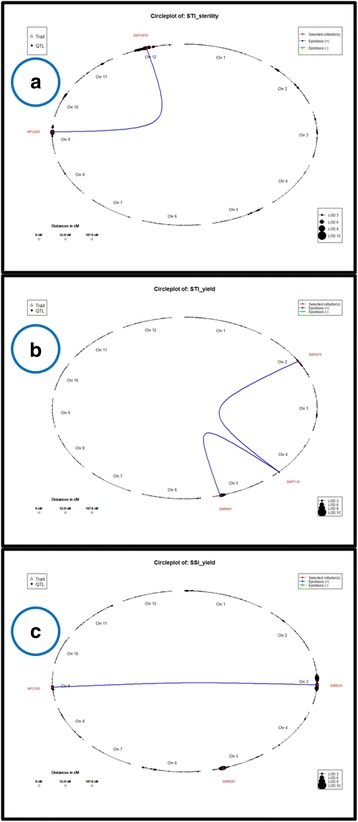
Epistatic interaction network between SNP marker loci. **a** STI for sterility (**b**). STI for yield (**c**). SSI for yield

### QTLs for Heat Tolerance Traits

Using inclusive composite interval mapping (ICIM) approach, a total of five QTLs for stress tolerance and stress susceptibility index for yield and percent spikelet sterility were identified (Fig. 4). One QTL each for SSI and STI of percent spikelet sterility and two and one QTLs for SSI and STI for yield, respectively were mapped on four different chromosomes, namely chromosomes 3, 5, 9, and 12 with phenotypic variation explained ranging from 6.37 to 21.29% (Table 4). *qSSIY5*.2 was the major QTL identified in this study for yield explaining 21.29% of the phenotypic variation while *qSTIPSS9.1* was the major QTL for percent spikelet sterility which explained 16.05% phenotypic variation. Except for *qSTIY5*.1, the heat susceptible parent IR64, contributed the heat sensitivity allele for both the spikelet sterility and yield QTLs. *qSTIY5.1* and *qSSIY5.1* are one and the same as they are present in the same genomic region. As expected, the tolerance allele of *qSSIY5.1* locus was contributed by N22 while the sensitivity allele was from IR64. Further, the additive effect of the trait enhancing allele from N22 was twice as that of IR64 (Table 4). Analysis of physical positions of the identified QTLs revealed that *qSTIY5.1/qSSIY5.1* was in a small interval of 331 kbp on chromosome 5. The other major QTL identified, *qSTIPSS9.1,* was also located in a small interval of 394 kbp on chromosome 9. The largest interval of 1067.5 kbp was for the QTL *qSSIY3*.1, which explained 6.45% of phenotypic variations of SSI for yield (Table 4). *qSSIPSS12*.1, a minor QTL for percent spikelet sterility was mapped between markers SNP14876 and SNP14892, explaining 6.37% of the phenotypic variation. As expected, the sterility enhancing allele for this QTL was from susceptible parent IR64. Thus, for all the four QTLs identified in this study, the heat tolerance allele was from the heat tolerant variety N22.

**Table 4:** QTLs for heat stress indices identified in RIL mapping population derived from N22 and IR64

Trait Name	QTL name	Chr.	Left Marker	Right Marker	Physical position (Mb)	Interval (Kb)	LOD	PVE (%)	Add
STI for % spikelet sterility	*qSTIPSS9.1*	9	SNP12393	SNP12417	16.75–17.14	393.828	4.21	16.05	-1.25
SSI_ for % spikelet sterility	*qSSIPSS12.1*	12	SNP14876	SNP14892	9.06–9.90	840.288	3.88	6.37	−0.33
STI for yield per plant	*qSTIY5.1*	5	SNP8377	SNP8401	25.45–25.79	331.586	4.55	9.01	0.07
SSI for yield per plant	*qSSIY3.1*	3	SNP5308	SNP5336	23.52–24.59	1067.507	4.04	6.45	−0.08
SSI for yield per plant	*qSSIY5.1*	5	SNP8377	SNP8401	25.45–25.79	331.586	3.51	21.29	−0.14

### Candidate Genes for Heat Tolerance Located in the QTL Intervals

The genes located in the four genomic regions for the identified QTLs were extracted (Additional file 2 **:** Table S1). Probable candidate genes for heat tolerance located in the major QTL interval *qSTIPSS9.1* for spikelet fertility were *PTC1*, glycosyltransferase, microtubule associated protein, annexin and *HSFs*. Similarly, candidate genes for heat tolerance index in the narrowed down QTL interval *qSTIY5.1/qSSIY5.2* included, trehalose synthase, trehalose-6-phosphate synthase, auxin response factor and calcineurin B-like protein-interacting protein kinase (CIPKs). In the QTL interval *qSSIPSS12.1*, 134 genes were identified including lipases, laccase, isoflavonereductase, cyclopropane-fatty-acyl-phospholipid synthase, *OGR1,* wall associated receptor kinase and pentatricopeptide protein coding genes. The *qSSIY3*.*1* QTL harboured 142 genes including, TFs, signalling genes and floral organ developmental genes, tesmin/TSO1, Crinkly4 receptor-like kinase, LIM domain containing protein gene and pectin methylesterase inhibitor coding gene. The majority of these genes are reported to be involved in pollen grain development, pollen tube growth and fertilization in rice.

### Digenic Interactions of QTLs for Heat Tolerance

Four significant digenic interactions involving seven SNPs were identified for three traits (Fig. 5) namely, STI for sterility and SSI and STI for yield on chromosomes 2, 3, 4, 5, 9 and 12 (Table 5). Interestingly, STI for yield had two digenic interactions involving a common SNP (7118) from a locus encoding dehydrogenase (LOC_Os04g52280). One of these interactions involved an SNP (8401) from the major and common QTL identified in the study, *qSTIY5.1/qSSIY5.2*. SNP8401 is present in a gene encoding WD40 domain, G-beta repeat domain containing protein (LOC_Os05g44320). All other SNPs showing epistasis were in uncharacterized expressed protein coding genes (Table 5).

**Table 5:** Epistatic interaction network

S. No.	Traits	Interaction	SNPs	Chr.	Locus	Physical position (bp)	Annotation
1	STI sterility	SNP12453-SNP14970	SNP12453	9	LOC_Os09g29160.1	17,733,252–17,731,555	expressed protein
			SNP14970	12	LOC_Os12g24090.1	13,706,157–13,705,072	expressed protein
2	STI yield	SNP8401-SNP7118	SNP8401	5	LOC_Os05g44320.1	25,793,638–25,788,753	WD domain, G-beta repeat domain containing protein, expressed
			SNP7118	4	LOC_Os04g52280.1	31,069,545–31,075,601	dehydrogenase, putative, expressed
		SNP7118-SNP3275	SNP3275	2	LOC_Os02g37380.1	22,580,613–22,579,160	expressed protein
3	SSI yield	SNP5239-SNP12183	SNP5239	3	LOC_Os03g38450.1	21,344,825–21,346,870	expressed protein
			SNP12183	9	LOC_Os09g18230.1	11,184,420–11,171,639	expressed protein

### Non-synonymous SNPs in the mapped QTL intervals

The maximum number of SNPs (71 SNPs in 26 genes) were observed in *qSSIPSS12*.1 genomic interval while the lowest number of SNPs (21 SNPs in 14 genes) were in *qSTIY5*.1 /*qSSIY5*.1 region (Table 6). Ty3-gypsy subclass retrotransposon protein encoding gene (LOC_Os12g17290) had the highest number of SNPs (8). Approximately, 77% of the observed substitutions were base transitions. SNPs were present in expressed genes, transcription factor coding genes, transposon related genes and protein and enzyme coding genes. *qSTIPSS9*.1 genomic region had 28 SNPs including 23 transitions and 5 transversions in 16 different genes. *qSSIPSS12*.1 interval had 71 SNPs in 26 genes including laccase precursor protein coding gene, expressed genes, Cyclopropane-fatty-acyl-phospholipid synthase coding genes and transposon related genes (Additional file 2 **:** Table S2).

**Table 6:** Non-synonymous SNPs between N22 and IR64 in the mapped QTL region for heat stress tolerance

S. No.	QTLs	No. of genes with SNPs	No. of SNPs	Non-synonymous SNPs between N22 and IR64	
				Transition	Transversion
1	*qstipss9.1*	16	28	23	5
2	*qssipss12.1*	26	71	58	13
3	*qstiy5.1 & qssiy5.2*	14	21	16	5
4	*qssiy3.1*	26	46	30	16

## Discussion

QTLs for heat tolerance have been mapped on different chromosomes of rice by different research groups during the last decade (Cao et al. 2003; Chen et al. 2008; Zhang et al. 2008, 2009; Jagadish et al. 2010; Xiao et al. 2011; Ye et al. 2012, 2015) (Additional file 2: Table S3). In the current study, using a reasonably large RIL population, high density SNP map and phenotyping under controlled facility, we identified four heat tolerant QTLs in rice, of which three were novel namely *qSTIPSS9.1, qSSIPSS12.1* and *qSSIY3.1*. Among these, *qSTIPSS9.1* was the major QTL for percent spikelet sterility. Further, we also identified a known major effect QTL, *qSSIY5*.1/*qSTIY5*.1 for both the indices of yield. Zhang et al. (2008) have reported this QTL in a RIL mapping population derived from a cross Zhongyouzao8 x Toyonishiki between SSR markers, RM405 and RM274 flanking a 23 Mb interval. In their study, this QTL explained 10.7% phenotypic variation for spikelet fertility under heat stress while it was for SSI/STI for yield in our study. Further, the QTL interval was narrowed down to a 331 kbp region comprising of 54 genes in our study. This was because earlier studies have used either SSR markers (maximum 264) or less than 300 SNP markers for mapping QTLs for heat tolerance whereas we have used more than 800 SNPs and 272 RILs to achieve a much higher resolution (Buu et al. 2014; Ye et al. 2012, 2015). Our ability to identify QTLs in such narrow intervals could be attributed to the use of 5K SNP array comprising of SNPs from abiotic stress responsive genes (Kumar et al. 2015). Some important candidate genes located in the high effect and minor QTLs identified in the present study are discussed below for their probable role in enhanced spikelet fertility and yield under heat stress.

There were 65 genes in the QTL region, *qSTIPSS9.1*, including transporters, transcription factors such as *HSF* (*OsHsfB4c*), *PHD*-finger domain containing TF (*PTC1*), *bHLH*, and *C2H2* zinc finger, transcriptional regulators, glycosyltransferase microtubule associated protein, and annexin (Additional file 2: Table S1). Tapetum, the innermost cell layer of the anther wall, plays a crucial role in anther development, microspore/pollen formation, and pollen wall formation. During late pollen development, tapetal degeneration triggered by an apoptosis-like process is essential for viable pollen formation (Li et al. 2006). *PERSISTENT TAPETAL CELL1* (*PTC1*) present in the *qSTIPSS9*.1 QTL region encodes a PHD-finger protein that controls programmed tapetal development and degradation to ensure functional pollen formation in rice (Li et al. 2011a, b). *PTC1* is expressed specifically in tapetal cells and microspores during anther development in stages 8 and 9 and initiates a typical apoptosis-like cell death, thereby ensuring proper pollen grain development (Li et al. 2011). Loss of function of *PTC1* displayed uncontrolled tapetal cell proliferation and swelling, delayed DNA fragmentation, and pollen wall development, causing complete male sterility (Li et al. 2011). Timely initiation of tapetal programmed cell death is essential for the regulated release of wall materials from the tapetum to the developing microspore including carbohydrate, lipid molecules, and other nutrients. This gene might be responsible for maintaining higher fertility in N22 under heat stress by timely initiation of PCD in N22 tapetal cell to ensure more fertile pollen grains than in the susceptible parent IR64. This gene otherwise named as a *tms9*–1/*OsMS1* is responsible for thermo-sensitive genic male sterility in *HengnongS*-1, one of the oldest and often-used TGMS line in *indica* two-line hybrid rice breeding programs in China (Qi et al. 2014). Also, *Arabidopsis thaliana MALE STERILITY1* (*MS1*) gene encodes for a protein homologous to the PHD-finger class of transcription factor and has been demonstrated to be involved in tapetal development and pollen wall biosynthesis (Yang et al. 2007).

Glycosyltransferase attaches a single or multiple sugars to different bio-molecules and highly expresses in mature pollen grains and is involved in mature pollen grain formation in rice (Moon et al. 2013). *GLYCOSYLTRANSFERASE1* (*OsGT1*) of rice present in *qSTIPSS9.1* is involved in pollen wall formation, especially, the exine and intine construction and pollen maturation. The *osgt1* mutant failed to produce mature pollen grains since its pollen had disrupted intine structure owing to low levels of starch and protein (Moon et al. 2013). Similarly, *uneven pattern of exine* 1 (*upex1*) gene of *Arabidopsis* encodes GT31 family glycosyltransferase in Arabidopsis and might be involved in galactosylation of arabinogalactan proteins (AGPs). The mutant of *UPEX1* exhibit defective and irregular exine pattern and suggests that primexine localized AGPs could play a role in sporopollenin adhesion and patterning in early microspore wall development (Li et al. 2016). HSFs are main players in imparting heat stress response by activating transcription of downstream genes including HSPs (Guo et al. 2008). *AtHsfB4* has a role in root development in Arabidopsis and involved in early stage of heat shock (Lohmann et al. 2004; Begum et al. 2013). A similar *OsHsf* has been identified in this major QTL on chromosome 9, which is yet to be characterized in rice.

Microtubule-associated proteins play a crucial role in the regulation of microtubule dynamics, and important for plant cell and organ development (Liu et al. 2013). The 65-kD microtubule-associated protein (MAP65) family member in Arabidopsis (*AtMAP65*–1) is ubiquitously expressed during the cell cycle and in all plant organs and tissues with the exception of anthers and petals (Smertenko et al. 2004). However, Microtubule-associated protein *MAP65*–1a (LOC_Os09g27700) of rice is expressed in anther and pistil. This might be indicative of its role in reproductive organ development in rice and hence is a good candidate for further studies in rice. Annexin functions to counteract oxidative stress, maintain cell redox homeostasis, and enhance drought tolerance (Szalonek 2015). Down-regulation of *Arabidopsis* annexin5 (*Ann5*) in transgenic *Ann5*-RNAi lines caused sterile pollen grains. Ann5 is involved in pollen grain development, germination and pollen tube growth through the promotion of endo-membrane trafficking modulated by calcium (Zhu et al. 2014). *TaAnn10* in wheat is highly expressed in floral bracts, pistil, anthers and immature endosperm and it correlates with anther development. But it fails to be induced by low temperature in thermosensitive genic male sterile lines, suggesting that specific down-regulation of *TaAnn10* is associated with cold induced male sterility in wheat (Xu et al. 2016). The relative expression levels of *TaAnn10* in the stamen strongly correlated with male fertility in recovery lines (Xu et al. 2016). One such annexin 10 (*OsANN10*/ LOC_Os09g27990) is present in the QTL interval *qSTIPSS9.1.*

A common response of organisms to drought, salinity, and temperature stresses is the accumulation of sugars and compatible solutes including trehalose. The increased trehalose accumulation correlates with elevated capacity for photosynthesis under both stress and non-stress conditions in rice (Garg et al. 2002). Trehalose-6-phosphate synthase (TPS) plays an important role in trehalose metabolism and signalling. Overexpression of the trehalose-6-phosphate synthase gene *OsTPS1* enhances the tolerance of rice seedling to cold, high salinity and drought stress without other significant phenotypic changes (Li et al. 2011). Similarly, the over-expression of trehalose-6-phosphate phosphatase in maize ears increases both kernel set and harvest index in drought stress condition. Increase in yield to the tune of 9% to 49% under non-drought or mild-drought conditions, and 31% -123% under more severe drought conditions, relative to yields from non-transgenic controls was observed (Nuccio et al. 2015). Similarly, trehalose concentration increased upon 4 h of heat stress at 40 °C and 4 days after cold stress at 4 °C in *Arabidopsis thaliana* (Kaplan et al. 2004). Over-expression of *ScTPS1* and *ScTPS2* under stress associated *rd29A* promoter provided protection against drought, salt, freezing, and heat stress (Miranda et al. 2007). Hence, trehalose synthase and trehalose phosphate synthase are a probable candidate genes underlying QTL *qSSIY5*.*1*/*qSTIY5*.*1* for yield under heat stress.

Our analysis for non-synonymous SNPs between the parents in the candidate genes like *PTC1*, *tms9*–1/*OsMS1*, *OsGT1*,*MAP65*–1a (LOC_Os09g27700), *OsANN10*, trehalose synthase, trehalose phosphate synthase, *OsCR4*, pectin methyl esterase and tesmin could not find allelic variants. This could be due to low coverage of the sequence data in either or both of the parents or lack of variation present in coding regions in the above genes. Further, the InDel polymorphism for these genes is not known. Alternatively, there could be variations in promoter, intron-exon junctions and UTR regions, which are not yet known. Hence, future effort is required to deep sequence these regions in the parents to identify polymorphisms, if any, in candidate genes. Nevertheless, the listed SNPs between N22 and IR64 can be utilised for fine mapping and functional validation of the QTLs.

The interaction network analysis showed evidence for the involvement of the major QTL, *qSSIY5*.1/*qSTIY5*.1, in digenic interactions, strengthening the role of this region in imparting heat tolerance. Though most of the SNPs involved in epistasis were in genes encoding uncharacterized expressed proteins, two SNPs were from known proteins coding genes namely, WD domain, G-beta repeat domain containing gene (LOC_Os05g44320) and dehydrogenase gene (LOC_Os04g52280). The WD40 protein is reported to play a role in diverse protein-protein interactions or protein-DNA interactions by acting as scaffolding molecule and promoting protein activity and thus functioning as a positive regulator of plant responses to various abiotic stresses such as salinity, osmotic and dehydration stress in plants (Mishra et al. 2012; Kong et al. 2015). Further, it is involved in various biological process, viz., signal transduction, gene transcriptional regulation, protein modifications, cytoskeleton assembly, vesicular trafficking, DNA damage and repair, cell death and cell cycle progression (Zhang and Zhang 2015).

## Conclusions

Present study using Nagina22/IR64 RIL mapping population and a 5K SNP genotyping chip, identified a major novel QTL *qSTIPSS9*.1 for reproductive stage heat tolerance in a 394 kbp region of rice chromosome 9. The study also confirmed the presence of a known major QTL for heat tolerance on chromosome 5 (*qSTIY5.1/qSSIY5.1*), which was narrowed down from 23 Mb in the original study to a much smaller interval of 331 kbp. This QTL was also involved in digenic interaction. Though the polymorphism survey in the candidate genes using the available data did not produce any trait linked variation, the SNPs identified could be useful in fine mapping. Further sequencing and functional validation is required for the identification of actual genes in these QTL regions responsible for the heat tolerance. Nonetheless, the two major QTLs identified here can be employed directly for crop improvement by marker assisted selection (MAS) after development of suitable scorable markers for breeding of high yielding heat tolerant rice varieties.

## Additional files

Additional file 1: Figure. S1. Atmospheric temperature at the experimental location during heat stress treatment. (TIFF 584 kb)

Additional file 2: Table S1. List of genes in the five QTL intervals identified. **Table S2.** Non-synonymous SNPs in the mapped QTL intervals. **Table S3.** Reported QTLs related to heat stress tolerance in rice. (XLSX 52 kb)
