# Supplementary figures and images for: High Resolution Mapping of QTLs for Heat Tolerance in Rice Using a 5K SNP Array
Source: Rice (N Y). 2017 Jun 5;10:28. doi: 10.1186/s12284-017-0167-0 (PMC5459777; doi:10.1186/s12284-017-0167-0)

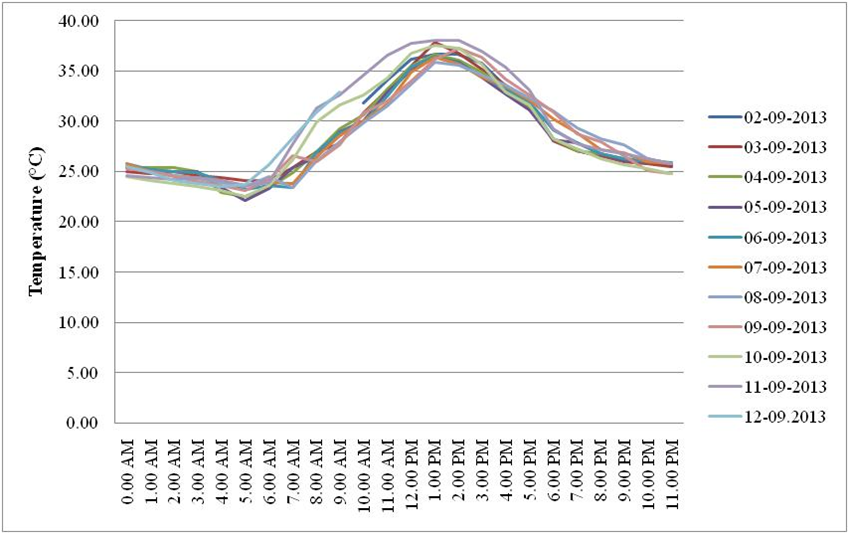

Supplement: Supplementary file 1 — Atmospheric temperature at the experimental location during heat stress treatment. (TIFF 584 kb) [file 12284_2017_167_MOESM1_ESM.tif]
